# Genetic influence of dopamine receptor, dopamine transporter, and nicotine metabolism on smoking cessation and nicotine dependence in a Japanese population

**DOI:** 10.1186/s12863-014-0151-2

**Published:** 2014-12-20

**Authors:** Masanori Ohmoto, Tatsuo Takahashi, Yoko Kubota, Shinjiro Kobayashi, Yasuhide Mitsumoto

**Affiliations:** Faculty of Pharmaceutical Sciences, Hokuriku University, Ho-3 Kanagawa-machi, Kanazawa, 920-1181 Japan

**Keywords:** *ANKK1/DRD2* TaqIA polymorphism, CYP2A6*4 polymorphism, Nicotine dependence *SLC6A3* VNTR polymorphism, Smoking cessation

## Abstract

**Background:**

This study investigated whether polymorphisms of the ankyrin repeat and kinase domain containing 1 gene (*ANKK1*), which is adjacent to the dopamine D2 receptor gene (*DRD2*), and the dopamine transporter (*SLC6A3*) and cytochrome P450 2A6 (*CYP2A6*) genes influence smoking cessation and nicotine dependence in a Japanese population. In 96 current and former smokers, genotyping frequencies for the *ANKK1*/*DRD2 Taq*IA, *SLC6A3* VNTR, and *CYP2A6* polymorphisms were subjected to chi-square analysis, and regression analyses were used to determine the association of the genotypes of current smokers with a Heavy Smoking Index, in addition to evaluating the effect of the subjects’ smoking history on the association.

**Results:**

Genotyping results suggested that nicotine dependence among current smokers homozygous for the *SLC6A3* 10r allele was lower than that of smokers carrying the minor alleles, and that the *CYP2A6* polymorphism might mediate this association. Furthermore, the age at which current smokers began smoking might moderate the association between their genetic polymorphisms and nicotine dependence.

**Conclusions:**

This study provides preliminary findings on the influence of genetic variants on the smoking phenotypes in a Japanese population.

## Background

Nicotine activates the mesolimbic dopaminergic system and mediates positive reinforcing reward effects, primarily by releasing dopamine in the nucleus accumbens [1]. Although smoking behaviour is affected by a combination of genetic and environmental factors, genetic factors are known to play a key role in some aspects of smoking behaviour [2]. The association of specific genetic variants with the molecular mechanisms underlying the behavioural phenotypes of nicotine addiction has been investigated extensively, with a focus on dopaminergic transmissions. The *Taq*IA polymorphism (rs1800497) of the ankyrin repeat and kinase domain containing 1 gene (*ANKK1*) [3]—adjacent to the dopamine D2 receptor gene (*DRD2*)—is known to be associated with smoking behaviour. Several surveys [4,5], predominantly with Caucasian subjects, have suggested that the A1 allele of this polymorphism increases the risk of smoking, whereas studies with Japanese subjects showed an association between the A2/A2 genotype and smoking risk [6,7]. We previously reviewed the effect of *ANKK1/DRD2* polymorphisms on smoking behaviour by considering the influence of ethnicity [8]. Our meta-analysis revealed a significant effect of *ANKK1/DRD2* polymorphisms on smoking cessation, which suggested that Caucasians carrying the A1 allele of the *Taq*1A polymorphism have a lower probability of smoking cessation than Asians do. It has been reported that the frequency of the A1 allele was higher in Americans (53–75%) than in Asians (11–58%) [9]. These significant ethnic differences in allele and genotype frequencies may be responsible for the inconsistent findings in previous studies on the role of the *Taq*1A *ANKK1/DRD2* polymorphism in the smoking behaviour of Caucasians and Asians.

The dopamine transporter (SLC6A3) terminates synaptic transmission by the rapid and specific reuptake of dopamine in the synaptic clefts. Lerman et al. [10] investigated the association of smoking risk with the variable number of tandem repeat (VNTR) polymorphisms (rs28363170) in *SLC6A3*, in combination with the *Taq*IA polymorphism, and found polymorphism–polymorphism interaction, in which individuals with the *SCL6A3* VNTR genotype that includes the 9-repeat (9r) allele were significantly less likely to be smokers, particularly if they also carried the *Taq*IA A2 allele. Sabol et al. [11] also demonstrated the significant effect of *SLC6A3* 9r genotypes on smoking cessation. However, other reports [12-14] did not replicate the initial positive results [10,11]. In studies of association between variant alleles of *ANKK1/DRD2* and *SLC6A3* and smoking, it has been suggested that the presence of the *ANKK1/DRD2 Taq*IA A1 allele along with the *SLC6A3* 9r allele increases cigarette craving that is induced by a stressor [15,16] and smoking reward and reinforcement by inducing a negative mood [17]. Furthermore, several reports [10,14,18] have suggested that compared to non-carriers, carriers of *SLC6A3* 9r allele have a lower risk of starting to smoke early.

The genetic effect of the pharmacokinetics of nicotine on the association between alterations in synaptic dopamine levels and smoking phenotypes has not been well documented to data. Nicotine in the blood is metabolised into cotinine mainly by cytochrome P450 (CYP) 2A6. One of the functional polymorphisms of *CYP2A6*, the *4 allele, is a particularly important polymorphic variant, with a gene deletion that is common in Asian populations [19]. It accounts for the majority of individuals with poor metabolism. Minematsu et al. [20] have reported that carriers of the *4 allele among Japanese smokers are more likely to be light rather than heavy smokers. Kubota et al. [21] also demonstrated that *CYP2A6* genotypes including the *4 allele are associated with nicotine dependence and withdrawal symptoms upon smoking cessation. These reports have suggested that smoking-related phenotypes may be influenced by altering the nicotine concentration in the brain as a sequel to reduced nicotine metabolism. We hypothesized that the association between the *ANKK1/DRD2* and *SLC6A3* polymorphisms and smoking-related phenotypes might be influenced by variants in *CYP2A6*.

In light of the lack of research in polymorphism–polymorphism interactions in the Japanese population, we here investigated whether the combined polymorphic variants of *SLC6A3* and *ANKK1/DRD2*, in the context of CYP2A6*4 genotypes, are associated with smoking-related phenotypes in a Japanese population, focussing on smoking cessation and nicotine dependence. Previous genetic association studies of these polymorphisms with smoking cessation and nicotine dependence are described briefly in Table 1. The present study also examined the effect of smoking history (age at which the participant began smoking and duration of smoking) on the association between SLC6A3, ANKK1/DRD2, and CYP2A6 polymorphisms and nicotine dependence.

**Table 1:** **Association between smoking behaviour and nicotine dependence and** ***ANKK1*** **/** ***DRD2 Taq*** **IA,** ***SLC6A3*** **VNTR, and** ***CYP2A6*** **polymorphisms**

**Study**	**Ethnicity**	**Samples**	**Association**	**Reference**
***ANKK1*** **/** ***DRD2 Taq*** ***IA polymorphism***				
Noble et al. (1994)	Caucasians	57 current smokers, 115 former smokers, and 182 non-smokers	Smoking subjects showed a significantly higher prevalence of the A1 allele compared to controls. Both past and current smokers demonstrated a significantly higher prevalence of the A1 allele than non-smokers did.	[4]
Comings et al. (1996)	Caucasians	312 smokers	There was a significant, inverse relationship between the prevalence of the A1 allele and the age of onset of smoking, and the maximum duration of time that smokers had been able to quit smoking on their own.	[5]
Batra et al. (2000)	Caucasians	110 heavy smokers and 60 light smokers	No significant findings	[22]
Bierut et al. (2000)	Caucasians	388 habitual smokers and 566 non-habitual smokers	No significant findings	[23]
Yoshida et al. (2001)	Japanese	77 current smokers, 57 former smokers, and 198 never smokers	Smoking appeared to be associated with the A2/A2 genotype.	[6]
Hamajima et al, (2002)	Japanese	226 current smokers, 133 former smokers, and 434 never smokers	Males with the A2/A2 genotype had a higher risk of being current smokers.	[7]
Johnstone et al. (2004)	Caucasians	752 smokers	At 1 week, the nicotine patch was more effective for smokers with the A1/A2 or A1/A1 genotypes than for those with the A2/A2 genotype; this was not the case at the 12-week flow up.	[24]
Morton et al. (2006)	Caucasians	1068 smokers, 213 non-smoking, and 1093 former smokers	Current smokers were more likely than former smokers to possess the A1 allele.	[25]
Connor et al. (2007)	Caucasians	84 smokers	Compared to carriers of the A2/A2 genotype, carriers of the A1/A1 or A1/A2 genotypes were characterised by higher levels of cigarette consumption.	[26]
***SLC6A3*** ***VNTR polymorphism***				
Lerman et al. (1999)	Caucasians (85%) African Americans (15%)	289 smokers and 233 non-smokers	Individuals with the 9r allele were significantly less likely to be smokers, particularly if they also carried the A2/A2 genotype. Smokers carrying the 9r allele genotype were also significantly less likely to have started smoking before 16 years of age and had prior smoking histories, indicating a longer period of prior smoking cessation.	[10]
Sabol et al. (1999)	Caucasians	164 current smokers and 111 former smokers	The 9r allele was associated with smoking cessation.	[11]
Jorm et al. (2000)	Caucasians	211 former smokers, 198 current smokers, and 452 non-smokers	No associations were found with either smoking initiation or smoking cessation.	[12]
Vandenbergh et al. (2002)	Caucasians	153 former smokers, 98 current smokers, 214 never smokers, and 114 non-smokers	Never smokers showed a higher prevalence of the 10r allele compared to current smokers. The frequency of the 10r allele in never-smokers (no cigarettes ever) was more than that in other smokers.	[13]
Perkins et al. (2008)	Caucasians	72 smoker	The increase in smoking amount owing to negative mood was associated with the A2/A2 allele and the 9r allele.	[17]
Laucht et al. (2008)	Caucasians	220 ever smokers (adolescents)	The A1 allele scored higher on nicotine dependence than their allelic counterparts. The intention to quit smoking was significantly lower in adolescents for the 10r/10r genotype.	[27]
Sieminska et al. (2009)	Caucasians	150 ever smokers and 158 never smokers	The abstinence periods during quitting attempts of carriers of the A1 allele were longer than those of non-carriers. The odds ratio for heavy smoking was higher in carriers of the A1 or 9r alleles compared to that in non-carriers. Compared to non-carriers, carriers of the 9r allele had a lower risk to start smoking before the age of 20 years.	[14]
***CYP2A6*** polymorphism (*4 allele)				
Tan et al. (2001)	Chinese	174 smokers and 152 non-smokers	The distribution of the *CYP2A6* genotype frequencies was not significantly different.	[28]
Loriot et al. (2001)	Caucasians	185 heavy smokers and 203 light smokers	No significant relationship between genetically impaired nicotine metabolism and cigarette consumption related and the presence of defective *CYP2A6* alleles (*2 and *4 alleles).	[29]
Ando et al. ( 2003)	Japanese	57 current smokers, 44 former smokers, and 139 never smokers	The proportion of never smokers among heterozygous carriers of the *4 allele was similar among subjects with the *1/*1 genotype. *CYP2A6* genotypes did not correlate either with the number of cigarettes smoked per day or with the age of smoking commencement.	[30]
Minematsu et al. (2003)	Japanese	92 current smokers, 111 former smokers, and 123 non-smoker	The percentage of subjects with a CYP2A6del (*4) allele was lower among heavy smokers than among light smokers or non-smokers and was lower among ex-smokers than among current smokers.	[20]
Fujieda et al. (2004)	Japanese	1094 patient (cancer) subjects and 611 healthy subjects	The amount of daily cigarette consumption in subjects who harboured the CYP2A6*4 allele was significantly less than that in subjects carrying the *1/*1 genotype.	[31]
Kubota et al. (2006)	Japanese	107 smokers	*CYP2A6* high-activity group (CYP2A6*1/*1, *1/*4, etc.) smoked the first cigarette of the day earlier than the low-activity group (CYP2A6*4/*4), indicating more marked nicotine dependence. Nicotine withdrawal symptoms were more serious during smoking cessation in the *CYP2A6* high-activity group.	[21]
Liu et al. (2011)	Chinese	970 current smokers and 358 former smokers	Poor metabolizers reported smoking fewer cigarettes per day, started smoking regularly at a later age, and smoked for a shorter duration than did normal metabolizers. However, poor metabolizers were less likely to quit smoking than normal metabolizers were.	[32]

## Results

The allele and genotype frequencies for the *SLC6A3*, *ANKK1/DRD2*, and *CYP2A6* polymorphisms in relation to smoking status for the 75 current and 21 former smokers are shown in Table 2. The distributions of the *SLC6A3* VNTR and *ANKK1/DRD2 Taq*IA genotypes in current smokers, former smokers, and all participants did not deviate from Hardy-Weinberg equilibrium (HWE) to any appreciable extent, as determined by chi-squared tests. The allele frequencies of the *SLC6A3* VNTR, *ANKK1/DRD2 Taq*IA, and CYP2A6*4 polymorphisms in all participants were similar to those from previous studies in Japanese populations [6,7,33,34]. Although the distribution of CYP2A6*4 genotypes in former smokers and all participants were different, the allele frequency of CYP2A6*4 in all participants were similar to those from a previous study [20].

**Table 2:** **Allele frequency profiles for** ***SLC6A3*** **,** ***ANKK1/DRD2*** **, and** ***CYP2A6*** **polymorphism genotypes for current and former smokers**

**Allele/Genotype**	**All** ^**a**^		**Current smoker**		**Former smoker**	
	**Number**	**%**	**Number**	**%**	**Number**	**%**
*SLC6A3*						
10r	172	89.6	136	90.7	36	85.7
9r	9	4.7	7	4.7	2	4.8
7r	8	4.2	6	4.0	2	4.8
6r	3	1.6	1	0.7	2	4.8
10r/10r	77	80.2	61	81.3	16	76.2
10r/9r	9	9.4	7	9.3	2	9.5
10r/7r	6	6.3	6	8.0	0	0.0
10r/6r	3	3.1	1	1.3	2	9.5
7r/7r	1	1.0	0	0.0	1	4.8
HWE^b^ *P*-value	0.96		0.37		0.31	
*ANKK1/DRD2*						
A2	112	58.3	91	60.7	21	50.0
A1	80	41.7	59	39.3	21	50.0
A2/A2	33	34.4	27	36.0	6	28.6
A2/A1	46	47.9	37	49.3	9	42.9
A1/A1	17	17.7	11	14.7	6	28.6
HWE^b^ *P*-value	0.89		0.77		0.51	
*CYP2A6*						
*1	164	85.4	126	84.0	38	90.5
*4	28	14.6	24	16.0	4	9.5
*1/*1	73	76.0	55	73.3	18	85.7
*1/*4	18	18.8	16	21.3	2	9.5
*4/*4	5	5.2	4	5.3	1	4.8
HWE^b^ *P*-value	0.02		0.07		0.04	

^a^Number of alleles or genotypes for combined current and former smokers; and ^b^Hardy-Weinberg equilibrium of genotype distributions of each polymorphism was tested for current smokers, former smokers, and the whole cohort.

The sex ratio and mean and standard deviation for age and smoking history of the participants, categorized by smoking status for each genotype of the *SLC6A3*, *ANKK1/DRD2*, and *CYP2A6* polymorphisms, are shown in Table 3. There was no significant difference in age and smoking histories of participants among genotypes.

**Table 3:** **Profiles of participants were categorized by smoking status for** ***SLC6A3*** **,** ***ANKK1/DRD2*** **, and** ***CYP2A6*** **polymorphism genotypes**

**(A) Current smoker**					
**Genotypes**	**Number**		**Age**	**Age at which**	**Duration of smoking**
	**Male**	**Female**		**participant began smoking**	
SLC6A3 10r/10r	57	4	31.51 ± 11.72	19.33 ± 2.01	11.82 ± 11.12
10r/# or #/#	12	2	36.93 ± 13.36	19.36 ± 1.84	16.43 ± 13.12
		*P*-value	0.119	0.855	0.361
ANKK1/DRD2 A2/A2	25	2	34.05 ± 13.89	19.67 ± 2.15	13.98 ± 13.99
A2/A1 or A1/A1	44	4	31.65 ± 11.09	19.15 ± 1.87	11.95 ± 10.05
		*P*-value	0.435	0.523	0.965
CYP2A6 *1/*1	50	5	32.58 ± 11.79	19.27 ± 2.09	13.05 ± 11.90
*1/*4 or *4/*4	20	1	31.90 ± 13.16	19.14 ± 2.31	11.24 ± 10.73
		*P*-value	0.716	0.846	0.654
**(B) Former smoker**					
**Genotypes**	**Number**		**Age**		
	**Male**	**Female**			
SLC6A3 10r/10r	15	1	48.69 ± 10.98		
10r/# or #/#	4	1	51.2 ± 6.98		
		*P*-value	0.901		
ANKK1/DRD2 A2/A2	6	0	50.667 ± 8.824		
A2/A1 or A1/A1	13	2	48.733 ± 10.760		
		*P*-value	0.697		
CYP2A6 *1/*1	16	2	49.44 ± 10.56		
*1/*4 or *4/*4	3	0	48.33 ± 8.02		
		*P*-value	0.840		

#DAT alleles with less than 10 repeats. *P*-value; the Mann–Whitney U test was conducted for participant age and smoking history of each genotype.

The gender ratio of the study population was skewed (see Table 2). Therefore, we performed analyses for the cohort as a whole (n = 96) as well as for the male subgroup only (n = 88). As shown in Table 4, subgrouping did not have an effect on detecting associations between the overall genotype frequencies and smoking status.

**Table 4:** **Odds ratios for the** ***SLC6A3*** **,** ***ANKK1/DRD2*** **, and** ***CYP2A6*** **genotypes in current and former smokers**

**Genotype**	**Number**	**OR (95% CI)**	***P*** **value**
*SLC6A3* 10r/10r	61/16, 57/15	1.362 (0.427–4.344), 1.27 (0.357–4.495)	0.831, 0.975
10r/# or #/#	14/5, 12/4		
*ANKK1/DRD2* A2/A2	27/6, 25/6	1.406 (0.488–4.050), 1.231 (0.416–3.642)	0.709, 0.917
A2/A1 or A1/A1	48/15, 44/13		
*CYP2A6* *1/*1	55/18, 49/20	0.458 (0.122–1.725), 0.459 (0.120–1.751)	0.376, 0.387
*1/*4 or *4/*4	20/3, 20/3		

Each analysis was performed for the whole cohort (the left side) and the male subgroup only (the right side). #DAT alleles with less than 10 repeats. Number, current smokers per former smokers; OR, odds ratio; and CI, confidence interval.

To assess whether the genotypes were associated with the Heavy Smoking Index (HSI) [35], as a measure of the degree of nicotine dependence, the HSI scores for current smokers were compared for each genotype, both in the whole cohort and in males only (Table 5). There was a significant association between the *SLC6A3* VNTR polymorphisms and the HSI score, whereas no associations were found between the *ANKK1/DRD2 Taq*IA and *CYP2A6* genotypes and the HSI score. The number of smokers with the CYP2A6*1/*1 genotype showing an HSI high-score was two-fold higher than that of smokers carrying the *4 allele.

**Table 5:** **Effect of genetic polymorphisms and smoking histories of participants on nicotine dependence in current smokers: Odds ratios for the** ***SLC6A3*** **,** ***ANKK1/DRD2*** **, and** ***CYP2A6*** **genotypes in current smokers with nicotine dependence**

**Genotype**	**Number**	**OR (95% CI)**	***P*** **value**
SLC6A3 10r/10r	9/52, 8/49	0.130 (0.036–0.464), 0.117 (0.030–0.459)	0.002, 0.003
10r/# or #/#	8/6, 7/5		
ANKK1/DRD2 A2/A2	4/23, 4/21	0.468 (0.136–1.615), 0.571 (0.161–2.032)	0.352, 0.570
A2/A1 or A1/A1	13/35, 11/33		
CYP2A6 *1/*1	15/39, 13/36	3.654 (0.757–17.634), 3.250 (0.661–15.979)	0.165, 0.235
*1/*4 or *4/*4	2/19, 2/18		

Each analysis was performed for the whole cohort (the left side) and the male subgroup only (the right side). #DAT alleles with less than 10 repeats. Number, numbers of subjects respectively indicated with high (≥ 4) per low scores (< 4) of HSI, Heavy Smoking Index (summary score of the number of cigarettes smoked per day and the time to the first cigarette of the day extracted from the Fagerstrom Test for Nicotine Dependence) in current smokers; OR, odds ratio; CI, confidence interval.

Moreover, we evaluated the effect of the *ANKK1/DRD2* and *CYP2A6* genotypes on the association between the *SLC6A3* VNTR polymorphism and the HSI score (Table 6). Regression analyses showed that the HSI score correlated better with the *SLC6A3* VNTR and *CYP2A6* genotypes than with the *SLC6A3* VNTR only in the total cohort (AIC value: 75.327). Regression analysis of the male subgroup only also showed a high correlation between the HSI score and the *SLC6A3* VNTR and *CYP2A6* genotypes, although this did not reach statistical significance (AIC value: 70.761; P = 0.018).

**Table 6:** **Effect of genetic polymorphisms and smoking histories of participants on nicotine dependence in current smokers: Regression analysis of the effect of combinations of genetic polymorphisms on nicotine dependence**

**Genes**	**R** ^**2**^	**AIC**	***P*** **-value**
*SLC6A3*	0.054, 0.048	77.940, 72.719	0.025, 0.040
*SLC6A3 + ANKK1/DRD2*	0.057, 0.260	78.660, 74.262	0.045, 0.098
*SLC6A3 + CYP2A6*	0.098, 0.087	75.327*, 70.761	0.009, 0.018

Each analysis was performed for the whole cohort (the left side) and the male subgroup only (the right side). Forward-selection regression began with the effect of the *SLC6A3* polymorphism alone. Variables were added one at a time to the model until no remaining variable produced a significant result. *SLC6A3*: input 1 or 0 for the 10r/10r or other genotype, respectively; *ANKK1/DRD2*: input 1 for the A2/A2 genotype, 0 for the A1/A2 or A1/A1 genotypes; *CYP2A6*: input 1 for the *1/*1 genotype, 0 for genotypes including the *4 allele. R^2^, squared multiple correlation coefficient adjusted for degrees of freedom; AIC, Akaike’s information criterion. *The appropriate model was selected on the basis of minimising AIC.

The proportion of HSI scores ≥4 for individuals with the *SLC6A3* 10r/10r genotype was lower than that of individuals with a single or no copy of the 10r allele, suggesting that the *CYP2A6* genotype might affect the relationship. We performed regression analyses to determine the effect of two variables in smoking histories on the association between nicotine dependence and genetic polymorphisms. As shown in Table 7, the HSI score was significantly correlated with the *SLC6A3* VNTR and *CYP2A6* genotypes when the age at which the participant began smoking was included as a variable in analysis of the whole cohort. Regression analysis of the male subgroup only (AIC value: 74.250), rather than that of the whole cohort (AIC value; 69.921), also showed a high correlation between the HSI score and a 3-variable combination (*SLC6A3* genotypes, *CYP2A6* genotypes, and age at which the participant began smoking), although this did not reach statistical significance (P = 0.014).

**Table 7:** **Effect of genetic polymorphisms and smoking histories of participants on nicotine dependence in current smokers: Regression analysis of the effect of smoking history on the association between genetic polymorphisms and nicotine dependence**

**Variable**	**R** ^**2**^	**AIC**	***P*** **-value**
*SLC6A3 + CYP2A6*	0.098, 0.087	75.327, 70.761	0.009, 0.018
*SLC6A3 + CYP2A6 + A*	0.133, 0.110	74.250*, 69.921	0.007, 0.014
*SLC6A3 + CYP2A6 + D*	0.127, 0.101	74.808, 70.615	0.009, 0.019

Each analysis was performed for the whole cohort (the left side) and the male subgroup only (the right side). Forward-selection regression was conducted with the effect of the *SLC6A3* and *CYP2A6* genes. Variables were added one at a time to the model until no remaining variable produced a significant result. A: age at which participant began smoking, D; duration of smoking. R^2^, squared multiple correlation coefficient adjusted for degrees of freedom; AIC, Akaike’s information criterion. *The appropriate model was selected on the basis of minimising AIC.

## Discussion

This study examined whether functional polymorphisms in *SLC6A3*, *ANKK1/DRD2*, and *CYP2A6* affect smoking cessation and nicotine dependence in a Japanese population. We found that current smokers with the *SLC6A3* 10r/10r genotype were more likely to have low nicotine dependence, based on HSI analysis, although the genotypic differences between current and former smokers were not significant for any of the *SLC6A3*, *ANKK1/DRD2*, and *CYP2A6* polymorphisms tested. Previous studies [36,37] suggested that the 9r allele enhanced the expression of the SLC6A3 protein, resulting in reduced postsynaptic dopamine activity. The 10r allele has been implicated in reduced SLC6A3 protein expression; thus, it might decrease the extent of nicotine dependence, by increasing the total amount of dopamine that is released into the synaptic cleft, thereby providing a greater reward from the dopaminergic effects of nicotine.

Regarding the association between the *SLC6A3* VNTR polymorphism and smoking cessation, initial studies [10,11] and a meta-analysis [38] have suggested that individuals carrying the 9r allele, rather than the more common 10r allele, had a greater likelihood of smoking cessation. However, these results have not been replicated [12,13], and the meta-analysis did not include Asian populations. Further studies are therefore needed to clarify the effects of the 9r and 10r alleles on smoking cessation.

As for the effect of the *SLC6A3* VNTR polymorphism on nicotine dependence, O’Gara et al. [39] reported a lack of association between the *SLC6A3* VNTR polymorphism and the HSI score for smokers attempting to quit by using either nicotine replacement therapy or bupropion. Nicotine dependence has been estimated using the Fagerstrom Test for Nicotine Dependence (FTND) [40]. De Leon et al. [41] suggested that use of the FTND in epidemiological surveys may lead to inaccurate conclusions, and that nicotine dependence should be measured only by the number of cigarettes smoked per day or the time to the first cigarette of the day. In addition, defining heavy smoking as more than 30 cigarettes per day would lead to underdiagnosis of individuals with high nicotine dependence. We therefore assessed nicotine dependence using the more accurate HSI and obtained significant results that suggested that low nicotine dependence was related to the 10r/10r genotype. Kozlowski et al. [42] suggested that their scales of nicotine dependence should be limited to predicting how heavily a person smokes rather than predicting the chances of quitting smoking. Thus, the differing relationships between smoking cessation and nicotine dependence with genetic influences are probably not contradictory.

Our finding suggests that variants in *CYP2A6* might affect the association of the VNTR *SLC6A3* polymorphism with nicotine dependence, although no significant association was found between the *CYP2A6* polymorphism and nicotine dependence. Because heavy smoking (high HSI score) was more frequent among individuals carrying the CYP2A6 *1 allele, these results might indicate an association between the *CYP2A6* polymorphism and high nicotine dependence. Kubota et al. [21] had previously demonstrated that the HSI score was significantly higher in the *CYP2A6* high-activity group carrying the *1 allele than in the low-activity group (homozygous for minor alleles, including *4). We found that current smokers with the 10r/10r genotype were more likely to have low nicotine dependence based on HSI analysis, although the genotypic differences between current and former smokers were not significant for any of the *SLC6A3*, *ANKK1/DRD2*, and *CYP2A6* polymorphisms.

Chronic exposure to a low concentration of nicotine is considered to desensitize nicotinic acetylcholine receptors significantly, which then turn over more slowly [43]. Individuals with high CYP2A6 activity may be able to maintain a low level of nicotine in the brain, which might influence dopaminergic activity via the nicotinic receptor, resulting in a craving for a short term, large dose of nicotine.

We speculate that the 10r/10r genotype might decrease expression of the SLC6A3 protein, which might result in a chronically high level of extracellular dopamine, protecting them from a craving for heavier smoking. The existence of a null allele in *CYP2A6* affects the related enzyme activity. It is possible that individuals carrying the *1 allele could inactivate nicotinic receptors constitutively by the high activity of CYP2A6, decreasing dopamine release into the synaptic clefts. *CYP2A6* polymorphisms might mediate the association between the 10r/10r genotype and low nicotine dependence.

We also determined the effect of smoking history on the relationship between nicotine dependence and genetic polymorphisms. Our results suggested that the age at which current smokers began smoking might moderate the effect of *SLC6A3* and *CYP2A6* polymorphisms on nicotine dependence. Individuals with the *SLC6A3* 9r genotypes were significantly less likely to have started smoking earlier [10,14]. A previous survey conducted on currently smoking adolescent subjects demonstrated that individuals homozygous for the 10r allele had a significantly lower intention to quit smoking than their allelic counterparts [18] did. Our findings suggested that the age of smoking initiation might be associated with nicotine dependence, under the influence of the *SLC6A3* VNTR polymorphism.

Our results showing an association between the *ANKK1/DRD2 Taq*IA polymorphism and smoking status were not consistent with previous data on Japanese males [6,7]. Previous studies have suggested that the A2/A2 genotype increased the risk of being a current smoker among the Japanese population, whereas studies with Caucasian subjects suggested that the A1 allele was associated with susceptibility to smoking [4,5]. A previous meta-analysis [44] suggested a lack of association between the *Taq*IA polymorphism and smoking behaviour and found evidence of strong heterogeneity between studies. No association of the HSI score with *Taq*IA polymorphism was observed in this study. The *Taq*IA polymorphism may thus not have a simple association with smoking status and nicotine dependence.

The *Taq*IA polymorphism was originally thought to be located in the 3′-untranslated region of *DRD2*, but recent evidence suggests that it lies within the region encoding the putative substrate-binding domain of ANKK1 [3]. The role of ANKK1 has not been fully elucidated, but the *Taq*IA polymorphism may be in linkage disequilibrium, in an ethnic group-specific manner, with unidentified polymorphisms in a neighbouring gene that functions in the signal transduction pathway and that has a stronger influence on dopamine reward processing.

There are several limitations to this study. First, the small sample size must be noted. The inconclusive results may have been the result of insufficient statistical power to detect associations with small effects. Because of the small sample size, we did not standardize the environmental factors in detail, which may have caused selection and confounding biases. Second, the accuracy of the self-reported questionnaire was not validated, and the screening test for nicotine dependence did not type participants into subtypes. Third, the molecular mechanisms underlying the associations between the *SLC6A3* VNTR and *ANKK1/DRD2 Taq*IA polymorphisms and smoking behaviour are uncertain and require clarification.

The degree to which our results can be generalized is not clear, but the present study provides a preliminary report in a Japanese population, and suggests that genetic studies on smoking should be based on ethnicity. Future large analyses on the multiple influences of polymorphism–polymorphism interactions, i.e. among the functional genetic polymorphisms of *SLC6A3*, *ANKK1/DRD2*, *CYP2A6*, and other related molecules, on smoking behaviour and nicotine dependence in different ethnic groups could address the problem of small sample size and lead to conclusions that are more reliable.

## Conclusions

The genotyping results suggest that nicotine dependence in current smokers who are homozygous for the *SLC6A3* 10r allele was lower than that in individuals carrying the minor alleles, and that *CYP2A6* polymorphisms might mediate this association. Furthermore, the age at which smokers begin smoking might moderate the association between their genetic polymorphisms and nicotine dependence. This study provides preliminary results regarding the effect of the *SLC6A3* VNTR, *ANKK1/DRD2 Taq*IA, and CYP2A6*4 polymorphisms on smoking cessation and nicotine dependence in a Japanese population.

## Methods

### Participants

Ninety-six Japanese ever-smokers were recruited from among the students, staff, and their siblings at Hokuriku University. The institutional review committee of Hokuriku University approved this study, and all participants gave their informed consent.

Participants were categorized as current smokers (n = 75, 69 males, 6 females, mean age: 32.52 ± 12.13 years) or former smokers (n = 21, 19 males, 2 females, mean age: 49.29 ± 10.07 years) if they had quit at least 1 year prior to the interview. The current smokers completed the FTND [40] as a self-reported measure of nicotine dependence and a lifetime history of cigarette smoking (the age at which they began smoking and the number of years they had smoked) was collected. Nicotine dependence was estimated by the HSI, which is based on two items extracted from the FTND: the number of cigarettes smoked per day and the time to the first cigarette of the day. A cut-off score of HSI ≥ 4 was used to categorize individuals as highly dependent on nicotine [41].

### Genotyping

Buccal swabs were collected from all participants and DNA was extracted with a DNA extraction kit (EPICENTRE® Biotechnologies, Madison, WI). The *SLC6A3* VNTR polymorphisms were amplified by PCR [33] and resolved on 1.5% agarose gels using positive controls obtained by direct DNA sequencing. To genotype the *Taq*IA polymorphism, the amplicons were digested with *Taq*I [4] and resolved on 2% agarose gels. The genotyping of CYP2A6*4 was performed by the PCR-RFLP method, using digestion with *Eco*81I [45].

### Statistical analyses

The genotypes of the polymorphisms were classified by the homozygosity of the major alleles as follows. *SLC6A3* VNTR: 10r/10r versus 10r/* or */*, where * refers to alleles with fewer than 10 repeats; *Taq*IA: A2/A2 versus A1/A2 or A1/A1; CYP2A6*4: *1/*1 versus *1/*4 or *4/*4. The VNTR, *Taq*IA, and CYP2A6*4 polymorphisms were tested for HWE in current smokers, former smokers, and the whole cohort.

The effect of the genetic polymorphisms on smoking cessation and the genotype frequency among the current and former smokers, and nicotine dependence estimated by the HSI score among current smokers, were examined. These analyses were conducted for both the whole cohort and male subjects only, because female participants accounted for only 8.3% of the cohort. Chi-squared analyses with the Yates correction were conducted to examine the association of genotype with smoking status and nicotine dependence. *P* < 0.01 and a 95% confidence interval (CI) that did not include a value of 1.0 were considered statistically significant. The associations were further expressed as odds ratios (OR) with a 95% CI.

We investigated the degree of nicotine dependence (the HSI score) among the current smokers generated by polymorphism–polymorphism interactions and the smoking histories of participants by two approaches using regression analysis. First, regression analyses were performed based on a method of forward-stepwise selection, by fixing the genetic polymorphism determined to be statistically significant by chi-squared analyses. Second, we performed forward-stepwise analyses using the two-variable combination of the age at which smoking began and the duration of smoking to determine the effect of smoking history on the relationship between nicotine dependence and the genetic polymorphism. The most appropriate model was selected based on Akaike’s information criterion (AIC). *P* < 0.01 was used as the cut-off for statistical significance.
